# Rules for Guidance in Chloroform Accidents Clinical Lecture on Anæmia

**Published:** 1862-04

**Authors:** 


### RULES FOR GUIDANCE IN CHLOROFORM ACCIDENTS.—By Dr. KIDD.

Reprinted from *Braithwaite.*

I. Young patients up to fifteen, and females bear chloroform best; there seems a general "tolerance" of chloroform in large surgical operations, probably from the hemorrhage keeping the blood moving and preventing engorgement of the cavities of the heart; in females the reflex system is more active than in males.

II. Anæsthesia under chloroform is not at all of the nature of imperfect oxygenation of the blood. But this imperfect oxygenation may be superadded and run parallel with anæsthesia, leading to fainting fits after administration; this is shown also by the plunging or violent efforts of the patient, from black blood in the muscles: every fresh concentrated vapor, from fresh additions to the inhaler, increases these muscular efforts, and always suggests the necessity of more air to the patient.

III. Where simple syncope is feared from fright, it is well to postpone the operation for a week, feeding the patient during this interval on meat and wine; the latter strengthens the muscles of the heart; it is well to bring such a patient under the chloroform, too, in a quiet ward rather than in the excitement of the operating-theatre.

IV. A passive, fixed, starting forward of the eyes, sudden contraction of the pupil, probably from spasm of the fifth nerve, and sndden change of the countenance, are the first signs of danger; the pulse may remain unchanged; chloroform should be kept warm (100° F.); this prevents coughing, which is also a dangerous symptom.

V. Simple syncope is much more dangerous than apnœa; it oc rs, however, less frequently.

VI. The operations where nearly all the accidents have occurred have been those for the removal of toe-nails, dead phalanges, tooth-drawing, strabismus, etc., operations on testis, reduction of dislocations,—all more or less connected with tendinous tissues.

VII. The right side of the heart, in apnœa cases, continues to beat long after the pulse stops. Probably while the iris of the patient contracts on approach of light we have a fair chance of waking up the entire heart; the right side of the heart will beat twelve or sixteen hours after the pulse at the wrist. Faradisation of the phrenic nerve or spine will set the heart in full action in animals through the renewed action of the respiratory muscles. Pulling out the tongue by forceps, so common hitherto in accidents, is a mistake; irritation of the nerves of deglutition stops the diaphragm.

VIII. Of 125 deaths, 54 occurred immediately before operation; 42 during small operations (syncope?); 25 from ether. The number of males in all returns seems to be exactly double that of females, though chloroform has been used in at least 30,000 cases of midwifery, and syncope is common in females. This leads to a suspicion that the deaths from chloroform are like railroad accidents in the management of cases.

IX. Vomiting, as in cataract cases, is best prevented by an aloetic purgative beforehand an hour or two; the stomach also to be empty at the time of operating, and the chloroform pushed well to deep anæsthesia.

X. The "cardiac syncope" of Snow is partly a post-mortem change. These are all cases of apnœa, or where the mischief arises essentially from fixture of the diaphragm. The heart, in fact, pulsates longer than the lung will receive or return the blood—hence the congestion, increased also by the efforts made at resuscitation by the surgeon himself.

XI. As to resuscitation, the means adopted at first should be as gentle as possible; a candle-wick gone out is to be blown in, not smothered by the *nimia diligentia.* Fanning the patient with cold fresh air is first in importance; the hands dabbed with cold water, which has a powerful influence in exciting the brachial and heart nerves. Too much cold water is not advisable. The patient should be brought at once into the open air, if summer, or into a very warm room, if winter; turned on his right side; the soles of the feet and interior of the ear tickled with a pen; his left arm held up, smacked with a wet towel, and that axilla and side of chest dashed with cold water; then artificial respiration tried by "up and down" pressure rather than "rotations." Two or three needles next stuck, where the omohyoid lies at the outer edge of the sterno-mastoid, so as to hit off, if possible, the phrenic nerve; then the *moist* pole of a Faradisation apparatus tried over the part, or along the spine; the other pole inserted under the floating ribs.

XII. It is probable, in the milder cases, the previous measures will succeed, especially in the instance of apnœa; but if, after five minutes' trial, little progress is made, then tracheotomy is to be done, and a small tube (a No. 10 india-rubber catheter) passed, and air blown gently into the lungs (a large tube causes emphysema). Some ammonia and warm water should be thrown into the rectum. In syncope cases, transfusion into a vein, of warm water with a little soda in it may wake up the heart. In apnœa (asphyxia as regards the heart, the right cavities gorged), opening a vein is useful. These measures should be continued for at least four to six hours, the body kept warm by hot blankets in a semi-recumbent position, the battery gases, and impure air of a crowd of students rigidly excluded; pure oxygen gas also to be avoided.

